# Supplementary material for: Effects of Preanalytical Sample Collection and Handling on Comprehensive Metabolite Measurements in Human Urine Biospecimens
Source: medRxiv. 2024 Jan 25:2024.01.24.24301735. Preprint. [Version 1] doi: 10.1101/2024.01.24.24301735 (PMC10896411; doi:10.1101/2024.01.24.24301735)
Supplement: Supplement 1 [file NIHPP2024.01.24.24301735v1-supplement-1.pdf]

medRxiv preprint doi: https://doi.org/10.1101/2024.01.24.24301735; this version posted January 25, 2024. The copyright holder for this preprint (which was not certified by peer review) is the author/funder, who has granted medRxiv a license to display the preprint in perpetuity.
This article is a US Government work. It is not subject to copyright under 17 USC 105 and is also made available for use under a CC0 license.

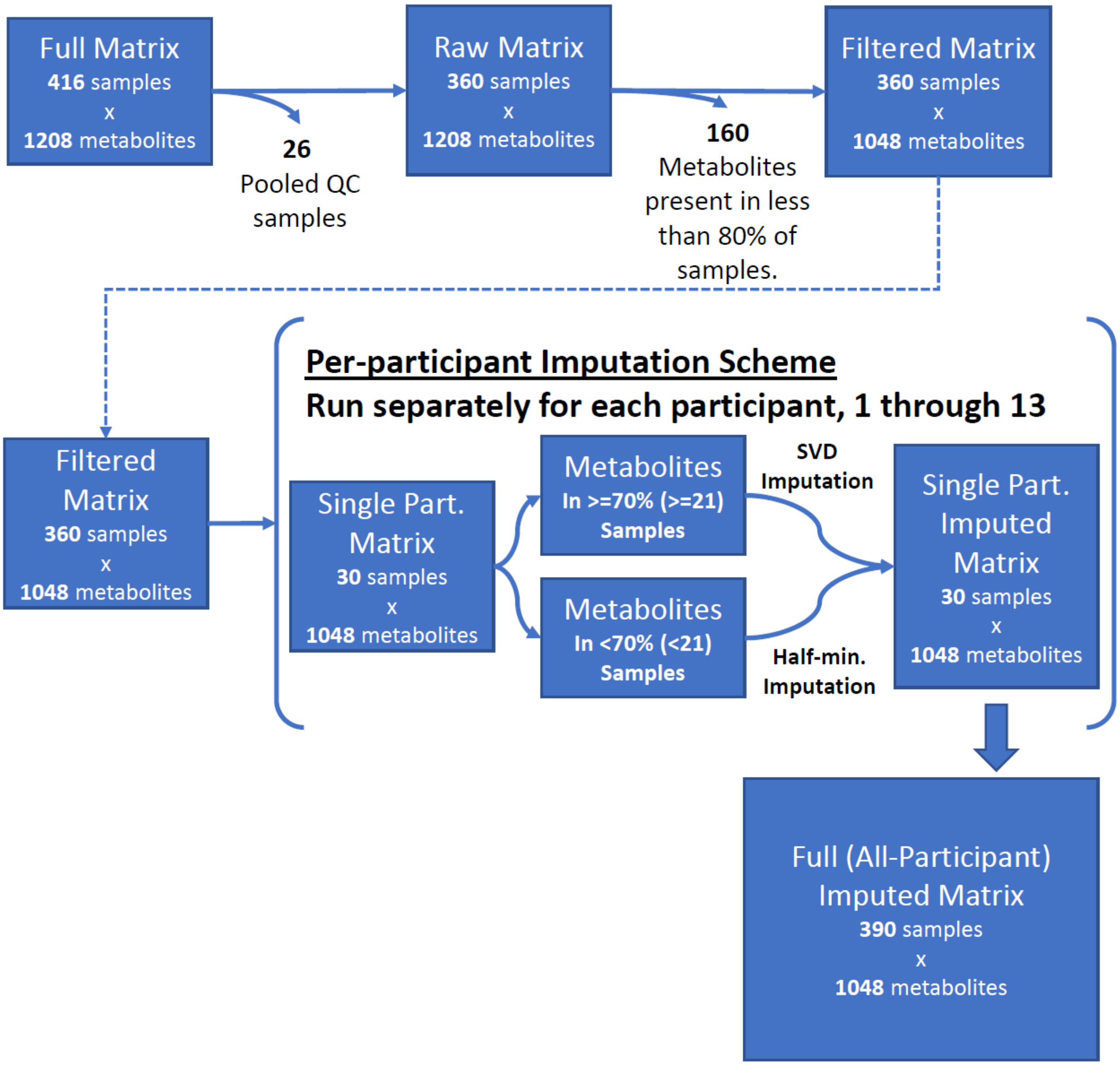

Supplementary Figure S1. Workflow delineating the data preprocessing steps.

medRxiv preprint doi: https://doi.org/10.1101/2024.01.24.24301735; this version posted January 25, 2024. The copyright holder for this preprint (which was not certified by peer review) is the author/funder, who has granted medRxiv a license to display the preprint in perpetuity.
This article is a US Government work. It is not subject to copyright under 17 USC 105 and is also made available for use under a CC0 license.

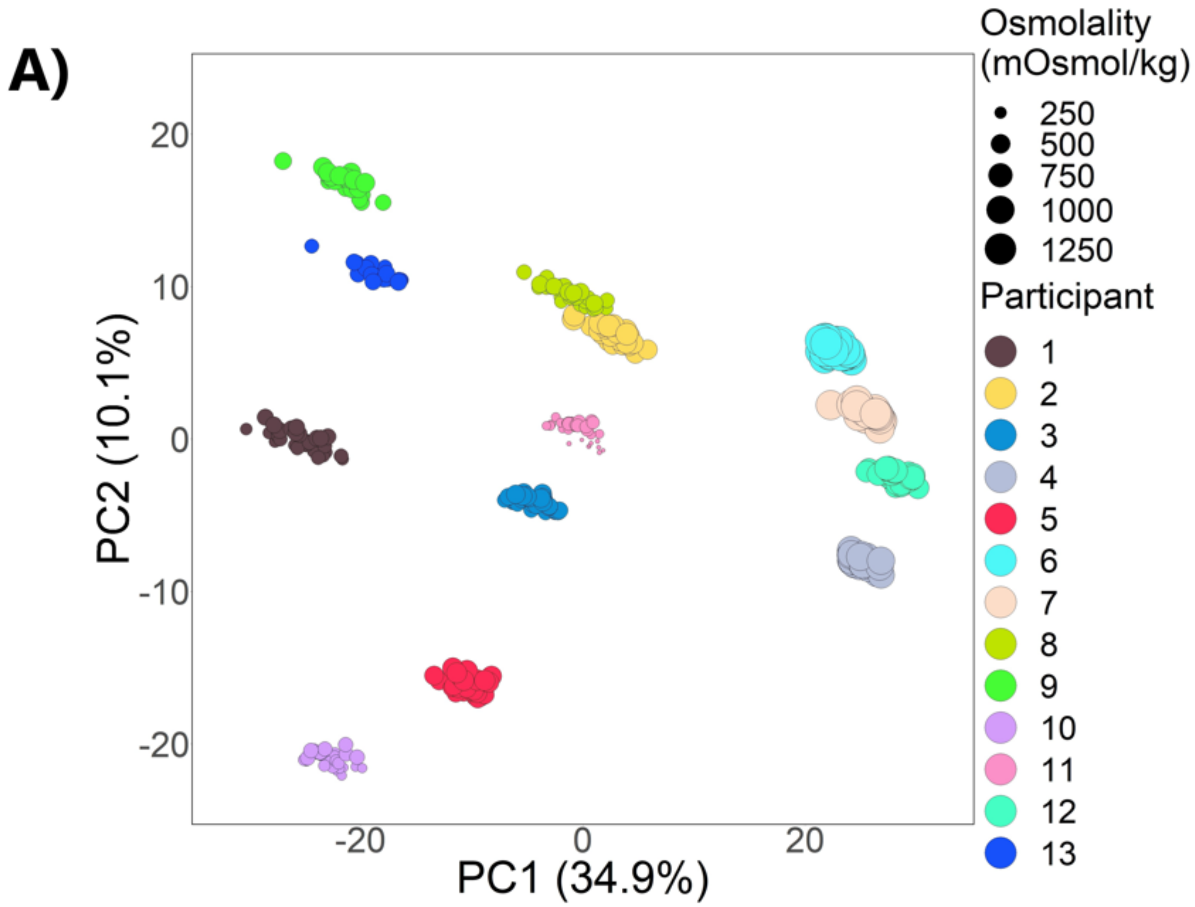

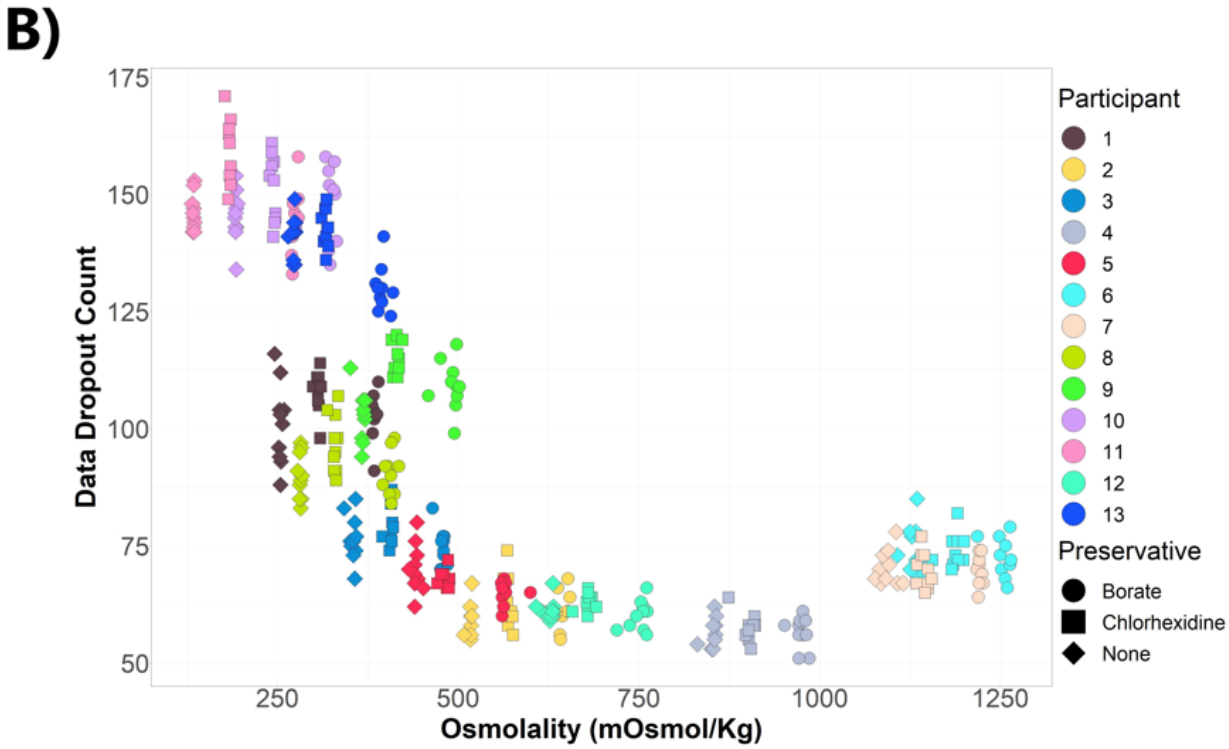

medRxiv preprint doi: https://doi.org/10.1101/2024.01.24.24301735; this version posted January 25, 2024. The copyright holder for this preprint (which was not certified by peer review) is the author/funder, who has granted medRxiv a license to display the preprint in perpetuity. This article is a US Government work. It is not subject to copyright under 17 USC 105 and is also made available for use under a CC0 license.

Supplementary Figure S2: Data quality assessment. A. Principal Components Analysis of our cohort metabolite abundance profiles. The input matrix included the log normalized abundances for 1,024 metabolites for each of the 390 samples (excluding QC samples). Colors are associated with participant ID. Point size relates to sample osmolality. B. Number of metabolite missing values for each sample as a function of sample osmolality. Preservative type is indicated by marker shape.

Supplementary Table S1. Total metabolites measured and those filtered out due to being missing in more than 80% of 390 samples. Super pathways are those provided by Metabolon.

Super Pathway	Total	Missing	Percent
No Annotation (X-mets)	537	56	10%
Xenobiotic	177	50	28%
Lipid	120	24	20%
Amino Acid	218	10	5%
Cofactors and Vitamins	35	8	23%
Nucleotide	52	5	10%
Peptide	24	5	21%
Carbohydrate	30	2	7%
Energy	15	0	0%
	1208	160	

Supplementary Table S2. Wilcoxon Rank Sum statistic comparing metabolite APD values between serum and urine metabolites for conditions common to both studies.

Treatment	Serum Mean	Urine Mean	Serum Median	Urine Median	pVal	Bonferroni-Adj pVal
**No Refrigeration vs.24 hr Refrigeration**	10.48	5.37	4.75	3.01	1.40E-10	9.82E-10
**No thaw vs 1x thaw on ice**	7.23	5.09	4.74	3.13	1.26E-09	8.81E-09
**No thaw vs 1x thaw in refrigerator**	9.84	6.09	6.04	3.75	1.86E-12	1.30E-11
**No thaw vs.1x room temperature thaw**	7.37	6.59	4.65	4.00	1.21E-01	8.46E-01
**No thaw vs 4x thaw on ice**	13.52	6.68	10.05	5.31	2.35E-36	1.64E-35
**No thaw vs 4x thaw in refrigerator**	15.56	6.50	7.25	4.18	9.88E-19	6.91E-18
**No thaw vs 4x thaw in room temperature**	7.23	5.75	5.54	3.85	5.64E-10	3.95E-09

medRxiv preprint doi: https://doi.org/10.1101/2024.01.24.24301735; this version posted January 25, 2024. The copyright holder for this preprint (which was not certified by peer review) is the author/funder, who has granted medRxiv a license to display the preprint in perpetuity.
This article is a US Government work. It is not subject to copyright under 17 USC 105 and is also made available for use under a CC0 license.

Supplementary Table S3. Number of false positive metabolites from the study simulation comparing serum to urine, for three levels of fraction of case samples having altered handling conditions, 0.05, 0.25 and 1.0.

	Proportion of Case Samples Handled Differently					
	Serum Study			Urine Study		
	**0.05**	**0.25**	**1**	**0.05**	**0.25**	**1**
**No Refrigeration vs.24 hr Refrigeration**	17	86	217	0	4	99
**No thaw vs 1x thaw on ice**	12	181	447	1	2	63
**No thaw vs 1x thaw in refrigerator**	22	205	459	1	4	77
**No thaw vs.1x room temperature thaw**	12	170	375	0	4	104
**No thaw vs 4x thaw on ice**	54	381	688	0	1	72
**No thaw vs 4x thaw in refrigerator**	41	196	386	1	3	67
**No thaw vs 4x thaw in room temperature**	8	92	244	0	3	76
**Average Number of False Positives**	**24**	**187**	**402**	**0**	**3**	**80**
